# Enhancing endoscopic spine surgery with intraoperative augmented reality: A case report

**DOI:** 10.1016/j.ijscr.2025.111342

**Published:** 2025-04-23

**Authors:** Don Young Park, Sofie Mei Park, Sohaib Hashmi, Yu-Po Lee, Nitin Bhatia, Michael Oh

**Affiliations:** a University of California Irvine, Department of Orthopaedic Surgery, 101 The City Drive South, Pavillion III, Building 29A, Orange, CA 92868, United States of America; b University of California Irvine, Department of Neurosurgery, 101 The City Drive South, Pavillion III, Building 29A, Orange, CA 92868, United States of America

**Keywords:** Augmented reality, Endoscopic spine surgery, Unilateral biportal endoscopy, Case report

## Abstract

**Introduction:**

Augmented reality (AR) has been recently implemented in spine surgery with current applications to visualize computer navigation while performing spinal instrumentation. Endoscopic spine surgery relies on high-definition video to perform the procedures. The combination of AR and endoscopic spine surgery can now be utilized to simultaneously display the endoscopic video and important clinical information to the surgeon during the procedure.

**Presentation of case:**

The patient is an 84-year-old male with low back pain and bilateral lower extremity pain and tingling that radiated to the posterior lower legs with the left side worse than the right. He had difficulty with walking long distances. He completed physical therapy with some improvement of his symptoms and gabapentin provided some relief. Physical examination was normal with no neurological deficits. Magnetic resonance imaging (MRI) of the lumbar spine demonstrated severe L4-5 stenosis. The patient elected to proceed with a biportal endoscopic L4-5 unilateral laminotomy and bilateral decompression (ULBD). The surgery was performed using Apple Vision Pro headset with the endoscopic video displayed within the headset simultaneously with the electronic medical record (EMR) and MRI images. The surgeon visualized the endoscopic video in a larger window as compared to the operating room monitor without perceptible lag or interruption. The procedure was completed without difficulty or complication. The patient was discharged on the same day and experienced significant clinical improvement over three months.

**Discussion:**

AR technology was successfully utilized for the first time to complete an endoscopic ULBD for the treatment of lumbar stenosis. The technology can provide useful clinical information such as the EMR and MRI images simultaneously with the endoscopic video. The large display can be positioned in front of the surgeon to optimize neck position and ergonomics. Novice learners and patients interested in endoscopic spine surgery can remotely experience the surgery since the surgery can be recorded from the surgeon’s perspective for educational purposes. Current AR headsets are relatively large and bulky, however, which may cause discomfort from the headset’s weight if used for long periods of time.

**Conclusion:**

AR technology can be effectively utilized in endoscopic spine surgery with several benefits to the surgeon. The technology can be used as an educational aid for surgeons learning the technique. As headsets become smaller, lighter, and more powerful, AR may become an important surgical tool, especially in endoscopic spine surgery.

## Highlights

• Augmented reality (AR) was utilized to successfully complete biportalendoscopic spine surgery.
• High-definition endoscopic video can be visualized with clinical data simultaneously in the AR view.
• AR technology can be an educational tool for new learners and patients who are interested in the procedure.
• Current forms of AR headsets are bulky and relatively heavy, which may deter the use for longer surgeries.
• AR may be combined with artificial intelligence and computer navigation to augment endoscopic spine surgery.

## 1 Introduction

Augmented reality (AR) in spine surgery has recently been implemented in clinical practice [1,2]. Current available AR clinical systems focus on intraoperative computer navigation for the placement of spinal instrumentation during fusion surgery [3,4]. The AR headsets project the navigation images collected from a 3D fluoroscopic scanner or a preoperative CT scan directly into the surgeon’s field of view. This application of AR may improve the surgeon’s ergonomics and maintains the surgeon’s attention to the surgical field, instead of looking away from the operative field at the navigation monitor [3]. However, in the current state, AR is utilized primarily for placing spinal instrumentation as an extension of existing computer navigation technology.

Endoscopic spine surgery has been growing in interest and utilization by spine surgeons as an ultra-minimally invasive technique to address common spinal pathology with reduced postoperative pain and faster recovery than conventional minimally-invasive or open techniques [5,6]. The endoscopic camera provides high-definition image quality that is highly magnified to visualize spinal anatomy, including neural structures. Typically, the endoscopic video is displayed on an operating room monitor or a monitor mounted on a tower that is directly relayed from the endoscopic camera. To our knowledge, no prior reports of using AR to view endoscopic video and simultaneously perform endoscopic spine surgery have been published. This case report describes the first use of AR by the operating surgeon to perform and complete a biportal endoscopic L4-5 laminotomy and bilateral decompression (ULBD) for lumbar spinal stenosis [7].

## 2 Presentation of case

### 2.1 Patient history and physical examination

The patient is an 84-year-old male with past medical history significant for type 2 diabetes mellitus, prostate cancer, hypertension, hypercholesterolemia, who experienced low back pain and bilateral lower extremity pain that radiated to the posterior lower legs with the left side worse than the right. He experienced tingling in the bilateral lower legs that was worse in the left side. His lower extremity pain and symptoms were worse than the low back pain. He had difficulty with walking distances longer than ½ mile and needed to rest and sit for relief. Bending forward alleviated his leg pain. He attempted physical therapy with some improvement of his symptoms and was taking gabapentin with some relief. The patient denied any bowel incontinence, bladder incontinence, or saddle anesthesia.

**Fig. 1:**
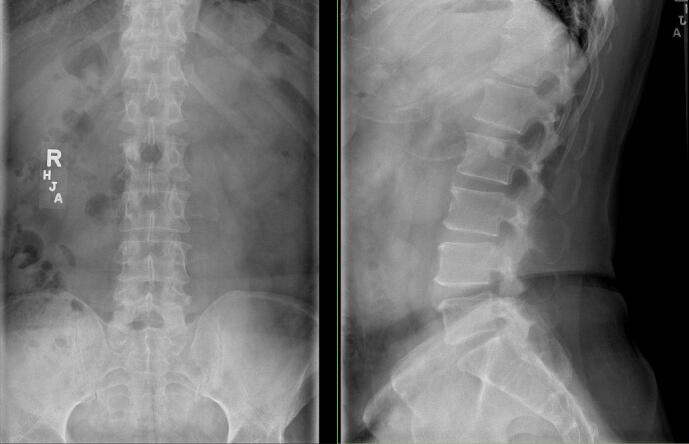
Anterior-posterior (AP) and lateral lumbar radiographs showing multilevel disc degeneration and lower lumbar spondylosis.

**Fig. 2:**
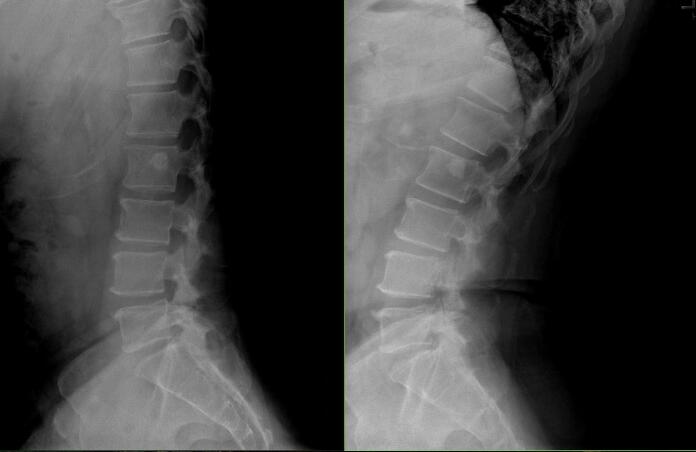
Flexion and extension lateral lumbar radiographs showing no change in alignment or instability.

**Fig. 3:**
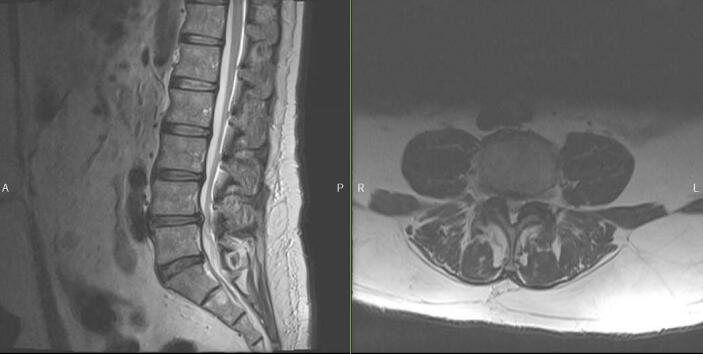
Sagittal and axial MRI images demonstrating severe L4-5 central stenosis from disc protrusion, ligamentum flavum hypertrophy, and facet hypertrophy.

His preoperative physical examination was unremarkable with a normal neurological examination. Radiographs of the lumbar spine demonstrated multilevel disc degeneration with no evidence of instability seen with upright flexion and extension lateral radiographs (Fig. 1, Fig. 2). MRI of the lumbar spine demonstrated similar findings with disc protrusions, facet hypertrophy, and ligamentum flavum hypertrophy contributing to L4-5 severe stenosis (Fig. 3).

Given that his pain and symptoms were significantly limiting his function and quality of life, the patient elected to proceed with surgery. His preoperative visual analog scale (VAS) back score was six, and the VAS leg score was nine. The preoperative Oswestry Disability Index (ODI) was 52 %. He elected to proceed with biportal endoscopic L4-5 unilateral laminotomy and bilateral decompression by the first author who has extensive experience in endoscopic spine surgery, having performed over 500 cases. Informed consent was obtained for the procedure, including the intraoperative use of AR during surgery as a means for visualizing the endoscopic video. The first author informed the patient that the endoscopic video would be simultaneously displayed on the overhead operating room monitors throughout the entire procedure. The patient agreed to proceed with the surgery utilizing augmented reality technology. This work has been reported in line with the SCARE criteria [8].

### 2.2 Augmented reality technology

**Fig. 4:**
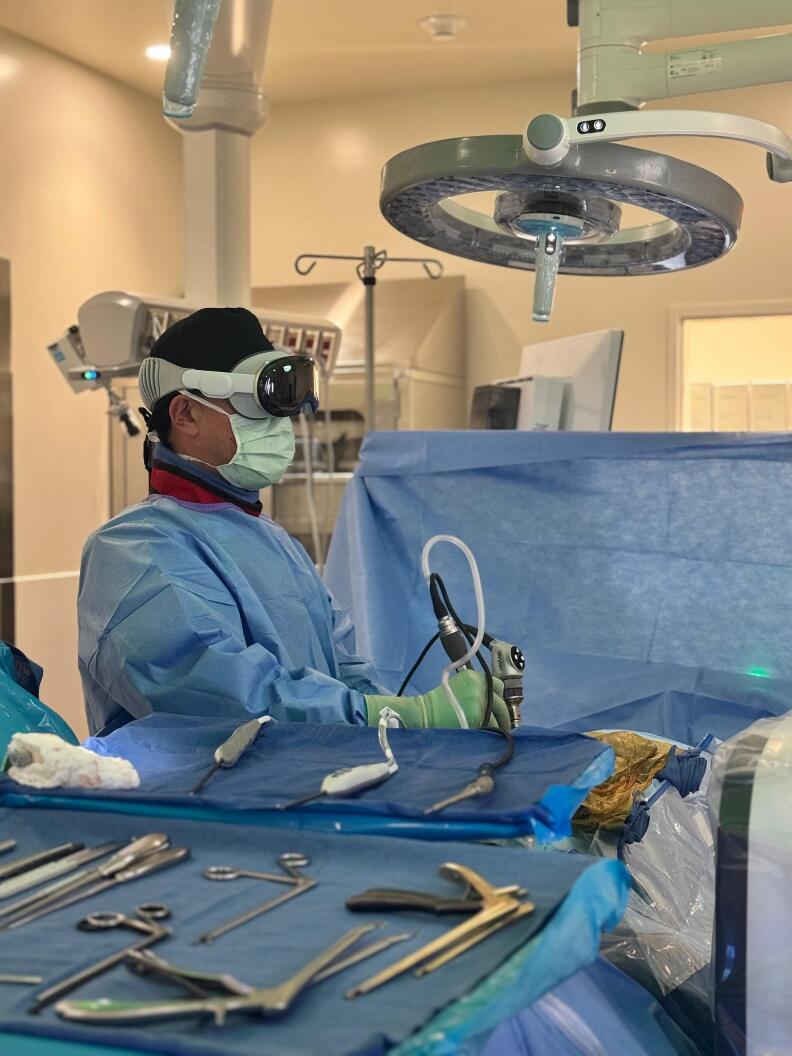
Surgical photograph depicting the surgeon performing biportal endoscopic spine surgery using the Apple Vision Pro headset.

The Apple Vision Pro headset was utilized for this case (Fig. 4). The Apple Vision Pro consists of a series of external cameras throughout the headset that record and project the data of the external environment into the two eye-sets within the headset [9]. Each eye-set independently projects at 5 K resolution to provide high quality video and data to the user wearing the headset. The high-definition endoscopic video was directly imported by HDMI cable from the endoscopic camera control unit in the operating room to a computer. The computer was placed immediately adjacent to the endoscopic camera control unit in the operating room. The Apple Vision Pro was then connected to the computer by WiFi wireless connection, and the applications were set up in the AR view on the headset. The external environment of the operating room with the operating table, instrument stands, staff, and operating room monitors was visible throughout the procedure with the AR headset.

**Fig. 5:**
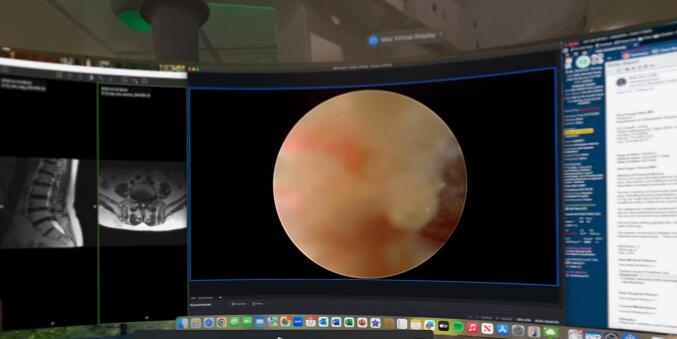
Intraoperative photograph depicting the endoscopic video in the center of the AR view, the preoperative MRI on the left of the AR view, and the clinical information from the EMR on the right of the AR view.

The relayed endoscopic video screen was displayed in the center of the AR view with the electronic medical record (EMR) and preoperative magnetic resonance imaging (MRI) images displayed to either side of the endoscopic video (Fig. 5). Real time high-definition endoscopic video was visualized through the AR headset, in addition to the operating room monitor adjacent to the AR view. This served to verify that the endoscopic video was accurate, and a backup system was in place if the AR system fails during the surgery. Due to the 5 K projection into the headset, as well as the 4 K video feed from the endoscopic camera, the endoscopic video was extremely high quality and was enlarged within the surgeon’s headset for greater detail.

### 2.3 Biportal endoscopic lumbar laminotomy and bilateral decompression

Once the AR headset was set up with the endoscopic video, EMR, and preoperative MRI on the AR view, the operating surgeon then scrubbed into the surgery (Video 1). After prepping and draping in the standard usual sterile fashion, fluoroscopy was used in the anterior-posterior projection to identify the midline and the left sided medial pedicle line, and these lines were drawn on the skin. The L4-5 level was identified using the lateral fluoroscopic projection and two portal incisions were planned and marked on the skin. The standard working portal measuring 7-8 mm in length and viewing portal measuring 4-5 mm in length were planned. The viewing portal is typically located cephalad for the endoscopic camera that is controlled with the left hand and the working portal is caudally located for the surgical instruments with a right-handed surgeon. Local anesthesia was administered, and the portals were made with an #11 blade scalpel. The procedure was performed as previously described [10].

The working space was created over the L4 lamina using a blunt tissue dissector through the working portal and the thoracodorsal fascia was dilated with a series of dilators. A semi-tubular outflow cannula was placed in the working portal to maintain an open endoscopic fluid outflow tract from the working space to the skin. The endoscopic camera was then introduced into the working space and triangulated with a radiofrequency probe. Endoscopic irrigation fluid was used to fill the working space, and hemostasis was performed with a radiofrequency probe. The bony landmarks were identified including the spinous process, lamina, the spinous process-lamina junction, and medial edge of the facet joint.

The laminotomy was then performed with a bone cutting shaver, starting at the spinous process-lamina junction (Video 2). The ligamentum flavum was exposed and released at the cephalad attachment on the L4 lamina. An “over-the-top” approach was then performed after drilling under the base of the spinous process to expose and release the contralateral ligamentum flavum (Video 3). The caudal attachments of the ligamentum flavum were then exposed and released with a drill and a Kerrison rongeur (Video 4). Once the lateral attachments were released with a Kerrison rongeur, the ligamentum flavum was resected to decompress the spinal canal (Video 5). Final decompression of the lateral recesses bilaterally was confirmed directly with the endoscope after resecting the osteophytes affecting the superior articulating processes on both sides (Video 6). At this point, hemostasis was obtained with a thrombin containing hemostatic agent and bone wax application over the exposed cancellous bone. A subfascial drain was placed within the laminotomy site and the endoscopic equipment was then removed. Excess irrigation fluid was removed, and the skin incisions were closed.

Throughout the procedure, there was no perceptible lag of the endoscopic video, interruption, or failure encountered between the AR system and the operating room monitor throughout the entire procedure. There were no intraoperative complications during the procedure.

The patient awoke from anesthesia uneventfully and recovered in the recovery room for several hours after surgery. After he had recovered sufficiently and ambulated, the surgical drain was removed, and the patient was discharged home with minimal back pain and elimination of the preoperative leg pain. It is standard practice of the first author that these surgeries are performed as outpatient same-day surgeries and the surgical drains are used until the time of discharge, at which point the drains are removed. If there is high drain output, then the drain would be maintained until the following day and the patient would be seen in clinic to remove the drain. Typically, the drain output has decreased significantly by this time. He was able to ambulate immediately after surgery and was started on outpatient physical therapy two weeks after surgery. Postoperatively, the patient had mild incisional pain in the low back, requiring acetaminophen and celecoxib without any opioid medications. His preoperative lower extremity pain had resolved after surgery and he exhibited no neurological deficits post-operatively. Three months after surgery, his VAS back score was 1, VAS leg score 0, and ODI score 12 %. There were no complications throughout his postoperative course.

## 3 Discussion

To our knowledge, this is the first case report in the scientific literature describing the successful utilization of an AR headset in an endoscopic spine surgery. The AR display was used intraoperatively to directly visualize endoscopic video throughout the procedure allowing successful completion of endoscopic spine surgery in a live patient. Throughout the duration of the procedure, there was no perceptible lag, interruption, or failure encountered with the endoscopic video while using the AR headset.

The AR headset offered several advantages to the surgeon. AR display allowed 5 K resolution endoscopic video with improved line-of-sight ergonomics in front of the surgeon’s eyes. In addition, AR allowed simultaneous access of relevant clinical data with the endoscopic video, such as the EMR clinical information and preoperative MRI. These could be immediately visualized by the surgeon without disturbance to the surgery, looking away from the surgical field or assistance of ancillary staff. The surgeon’s full attention can be maintained to the surgery at hand, making the surgery more efficient and safe.

However, there are several disadvantages of the Apple Vision Pro headset, such as the headset weight of 600–650 g, the bulkiness of the headset, and the relatively short battery life of approximately 2 h, In order to employ AR technology in longer surgical procedures, headsets must be smaller and lighter for improved surgeon comfort and the battery life should be longer for the prolonged surgeries. The headsets can be effectively used for training new surgeons since they can watch and learn the steps of the surgery from the point of view of the operating surgeon. However, the AR headsets should only be used during surgery by expert surgeons who have mastered endoscopic spine surgery with conventional viewing monitors and equipment. One of the risks discussed with the patient was that the headset could fail during surgery and there could be significant lag between the actual endoscopic video and the video seen on the Apple Vision Pro. It was explained to the patient that the operating room monitor would be visible at all times simultaneously with the AR view and if there was any failure or significant lag, then the conventional OR monitor would only be used. There was no failure or significant lag at any point and the AR headset was used throughout the entire procedure.

AR technology has already been utilized in spine surgery as an extension of computer navigation [[1], [2], [3], [4]]. In this application, intraoperative 3D fluoroscopic scan or preoperative CT scan can be projected on the AR headset and displayed over the patient’s anatomy to place spinal instrumentation such as pedicle screws for spinal fixation. Both cadaveric and clinical studies investigating the use of AR in spinal instrumentation have demonstrated similar accuracy and precision as conventional computer navigation systems [3,4,11,12]. Other applications for AR and virtual reality (VR) include educational applications for studying and understanding spinal and neurosurgical anatomy as well as training novice learners how to perform procedures such as lumbar punctures and even brain tumor removal [13,14]. Researchers are currently investigating how artificial intelligence (AI) can be incorporated into AR and spine surgery, although most are feasibility studies at this point [15,16].

In the near future, AR headsets will become smaller, lighter, and more ergonomic as the technology advances, and the applicability of AR in spine surgery would become more widespread. At this point, the ergonomic advantage of AR headsets is apparent with surgeons maintaining their focus on the surgical field rather than turning their heads and even their bodies toward the viewing monitor, which is typically some distance away from the surgical field. As seen in this case report, AR technology can enlarge the endoscopic video to a great degree for enhanced visualization and detail of the surgical anatomy, while simultaneously displaying other important clinical data to the surgeon in real-time. This access to critical clinical information enables the surgeon to make clinical decisions more efficiently during surgery.

One day soon, AI technology may be incorporated to display “smart” no fly-zones on the endoscopic AR video based on preoperative imaging and planning. AI may prompt the surgeon on the AR headset on the next best surgical move with a particular surgical instrument, based on millions of possible combinations for the most efficient and safest surgery. Computer navigation can be incorporated so that the navigation data is displayed on the AR headset with the other clinical information. All of this data can be recorded by the AR headset so that new learners and even patients can see what the surgeon sees throughout the surgery, which can be helpful in learning about the procedures [17]. Over time, a library of AR recorded videos can be collected with various types of endoscopic spine surgeries for educational purposes.

## 4 Conclusion

AR technology is currently being utilized in spine surgery as an extension of computer navigation for placement of spinal instrumentation. Educational applications of AR technology can help teach the steps of spine surgery to new learners and even patients prior to live surgery. We described the first case report of AR technology successfully used to display high-definition endoscopic video directly to the surgeon during endoscopic spine surgery. The time is near when AR will be successfully combined with AI, computer navigation, and other relevant clinical data so that the surgeon have them at his fingertips during surgery. In the current form, however, the bulk and weight of the AR headsets may limit the widespread applicability of the technology.

The following are the supplementary data related to this article.

Video 1 Video of the surgeon after scrubbing into the surgery and starting surgery while utilizing the Apple Vision Pro headset. Video 1

Video 2 Intraoperative video depicting the start of the laminotomy using the bone cutting shaver. Video 2

Video 3 Intraoperative video depicting the bone cutting shaver performing the “over the top” portion of the procedure, exposing the contralateral ligamentum flavum and releasing it for later removal. Video 3

Video 4 Intraoperative video depicting the release and removal of the ipsilateral ligamentum flavum. Video 4

Video 5 Intraoperative video depicting the release and removal of the contralateral ligamentum flavum. Video 5

Video 6 Intraoperative video depicting the final decompression of both sides of the spinal canal. Video 6

## Consent

Written informed consent was obtained from the patient for publication of this case report and accompanying images. A copy of the written consent is available for review by the Editor-in-Chief of this journal on request.

## Ethical approval

This work was approved by the Institutional Review Board.

## Funding

None.

## Author contribution

Don Young Park: Concept and design, paper writing, editing.

Sofie Mei Park: Paper writing, editing, review.

Sohaib Hashmi: Editing, review.

Hao-Hua Wu: Editing, review.

Hansen Bow: Editing, review.

Yu-Po Lee: Editing, review.

Nitin Bhatia: Editing, review.

Michael Oh: Editing, review.

## Guarantor

Don Young Park.

## Research registration number

None.

## Conflict of interest statement

There are conflicts of interest that are disclosed by the authors.
